# Comparative Study of Chaga (*Inonotus obliquus*) Dietary Supplements Using Complementary Analytical Techniques

**DOI:** 10.3390/ijms26072970

**Published:** 2025-03-25

**Authors:** Coleton Windsor, Anna E. Kreynes, Jeff S. Chilton, William A. Chioffi, Arun Krishnamurthy, Melissa Ishii

**Affiliations:** 1 North American Reishi, Ltd. D.ba Nammex, Box 1780, Gibsons, BC V0N 1V0, Canada; coleton@nammex.com (C.W.); bill@nammex.com (W.A.C.);; 2 Purity-IQ, Purity-IQ Inc., Suite 102, 150-Research Lane, Guelph, ON N1G 4T2, Canada; arun.krishnamurthy@purity-iq.com

**Keywords:** mycelium, fermented grain adulteration, triterpenoids, inotodiol, fungi, melanin, polysaccharides, chromatography, quality control, botanical authenticity

## Abstract

Chaga (*Inonotus obliquus*) is an increasingly used natural product in botanical dietary supplements, valued for its bioactive compounds. However, inconsistent standardized analytical methods raise concerns over product authenticity, mislabeling, and quality control. This study employs a multi-analytical approach to differentiate wildcrafted chaga canker from North American chaga dietary supplements, particularly those containing mycelia fermented grain products. High-Performance Thin-Layer Chromatography (HPTLC), Liquid Chromatography with Evaporative Light Scattering Detection (LC-ELSD) or Photo/Diode Array Detection (LC-PDA/DAD), Liquid Chromatography-Quadrupole Time-of-Flight Mass Spectrometry (LC-QToF-MS), Nuclear Magnetic Resonance (NMR) spectroscopy, UV-Vis spectrophotometry, and iodine-starch assays were used to evaluate key markers, including triterpenoids, polysaccharides, and melanin. Whole chaga canker contained triterpenoids (inotodiol, trametenolic acid) and phenolics, like osmundacetone, while melanin absorbance at 500 nm differentiated it from fermented grain products. β-Glucan quantification and iodine-starch assays confirmed starch-rich composition in fermented grains and its absence in authentic chaga canker. NMR fingerprinting and LC-QToF-MS metabolomics demonstrated stark compositional deviations between wildcrafted chaga canker, *I. obliquus* mycelium, and fermented grain products. By integrating complementary techniques, we establish a framework that can reliably distinguish genuine chaga canker from misrepresented products, ensuring consumer safety and fostering trust in the functional mushroom, canker, and mycelium markets.

## 1. Introduction

Chaga is the common name for the sterile canker that forms on birch trees (*Betula* spp.), such as paper birch (*Betula papyrifera*), as the tree’s response to infection by the pathogenic fungus *Inonotus obliquus* (*I. obliquus*) (Pers. ex Fr.) Pilát. Chaga is characterized by a black, cracked outer layer containing melanin [1] and a rusty-brown, streaked inner tissue [2,3]. The actual fertile fruiting body of *I. obliquus*, by contrast, forms under detached bark on dead or fallen trees as a grayish, resupinate mass up to 3–4 m long and 40–50 cm wide, with downward-cascading pores that release spores [4].

Chaga is commonly found in the northern circumpolar region, as *Inonotus* parasitizes birch and other hardwood species such as alder (*Alnus*), beech (*Fagus*), and ash (*Fraxinus*). Over decades, *I. obliquus* mycelia spread within the host tree, degrading cellulose, hemicellulose and lignin, causing heartwood decay. Infected trees continue to grow but typically have a shorter lifespan, with British Columbia foresters estimating a 50–100% reduction in timber value due to extensive decay from a single canker [2]. All chaga is harvested wild (wildcrafted), and currently no chaga has been successfully cultivated.

Chaga contains approximately 10% fungal mycelium, with the remainder consisting of decayed woody tissue [5]. This distinction is further supported by the low levels of fungal-specific markers, such as ergosterol and inotodiol, observed in chaga [5]. True sclerotia serve as resting structures for fungi, formed underground and capable of producing fruiting bodies under favorable conditions [6]. Chaga, in contrast, is a visible manifestation of fungal infection in the tree trunk.

The use of chaga in traditional medicine spans indigenous cultures in North America and Eurasia, where it has been employed for its alleged anti-inflammatory, antimicrobial, and antioxidant properties [7,8,9,10]. Today, chaga is highly regarded for its health benefits, including immune modulation [11,12,13], anti-inflammatory [5,14,15,16,17], antioxidant [5,17,18,19,20], antimicrobial [5,21] and antidiabetic effects [17,22,23,24,25] and potential anticancer properties [5,9,10,13,15,17,19,21,26,27,28,29,30,31,32,33,34,35,36,37]. These medicinal properties can be attributed to several classes of biologically active compounds found in chaga, including phenolics [5,20], sterols [15], polysaccharides [38,39,40], triterpenes [10,15,18,30,32,35,38], and melanins [1,8,24,38,41]. While many of these compounds can be found in a variety of other organisms, some are unique to chaga and therefore can be used as molecular markers for product authentication [42].

As consumer demand for chaga-based supplements rises, concerns have emerged over quality control, mislabeling, and authenticity. A major issue is the misrepresentation of mycelia fermented grain products as authentic chaga, despite their fundamental differences in composition and bioactivity. The differences between authentic mushroom products and mycelia-fermented grain were previously demonstrated with Reishi (*Ganoderma lucidum*), where the presence of starch and the absence of important secondary metabolites characteristic of Reishi were noted in many commercial products [43]. To address this issue of authenticity, we employ a multi-tiered analytical strategy combining simple assays and advanced instrumentation to differentiate wildcrafted chaga from fermented grain substitutes.

The polysaccharide profiles of fungi differ dramatically from those of grain: grain-based products are high in starch (⍺-glucans), with a small amount of β-glucans, and in some cases cellulose, while fungi are typically high in β-glucans, and devoid of starch. The β-glucan structure of chaga has been previously characterized as β-(1,3) with β-(1,6) branching [40,44], whereas cereal β-glucans (e.g., oats, barley) contain β-(1,3) and β-(1,4) linkages [45,46,47]. In this study, we use Lugol’s solution (I_2_/KI) to detect starch and enzymatic assays to quantify ⍺-and β-glucans in different chaga and commercial samples. Additionally, we employ UV-Vis and IR spectroscopy to detect melanin, an important polyphenolic macromolecule and antioxidant that gives chaga its distinct dark color [1,48]. 

To evaluate secondary metabolites, HPTLC, and LC-ELSD/UV are used to screen for key markers, including lanosterol-derived triterpenes and phenolic compounds. These findings are corroborated by LC-QToF-MS and ^1^H NMR spectroscopy, which provide detailed metabolomic comparisons. Principal component analysis (PCA) and Linear Discriminant Analysis (LDA) of these datasets reveal critical compositional differences between authentic chaga canker, pure mycelium, and fermented grain products.

By integrating complementary techniques, each targeting distinct chemical classes (polysaccharides, melanin, triterpenoids), this study establishes a robust framework to authenticate chaga supplements, addressing both quality control gaps and consumer safety concerns in the functional fungi market. This represents the first application of a multi-analytical approach, combining HPTLC, LC-QToF-MS, NMR, UV-Vis, and iodine-starch assays to differentiate wildcrafted chaga canker from mycelia fermented grain products. By identifying chaga-specific biomarkers, this study fills a critical gap in quality control for dietary supplements, ensuring product integrity, and regulatory compliance.

## 2. Results

### 2.1. Physical Appearance of Whole Chaga and Commercial Products

**Figure 1:**
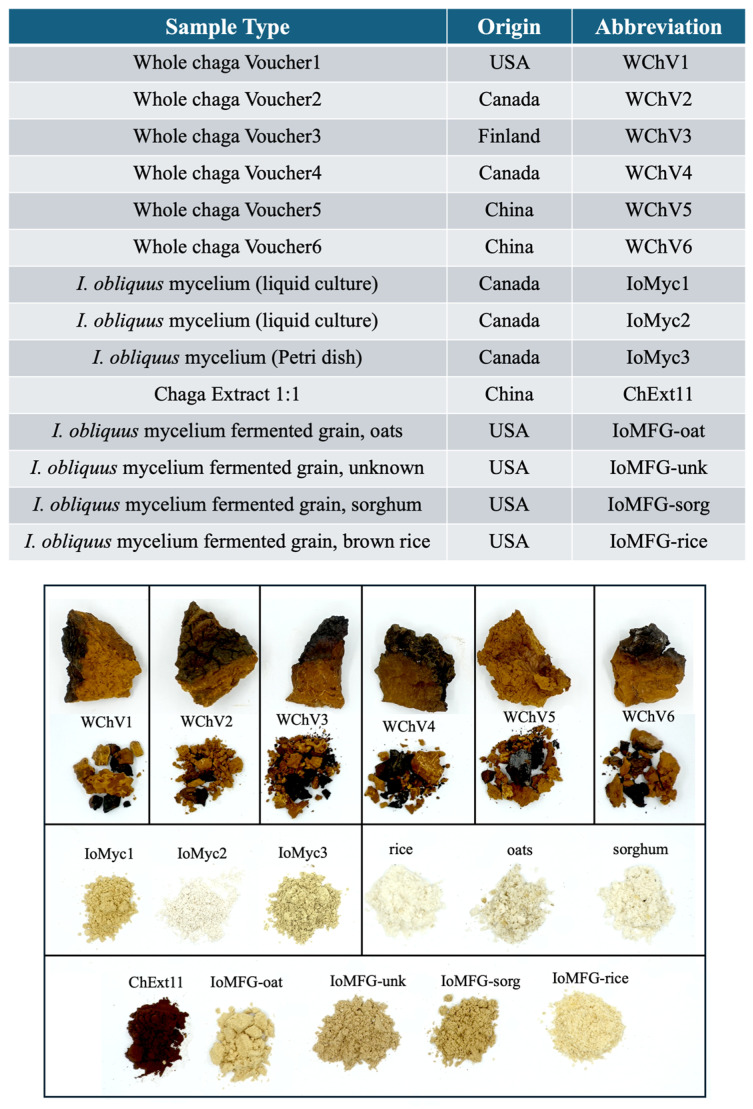
Samples used in this study. Top: summary of sample codes assigned to chaga vouchers and commercial products. Bottom: photographs of the sample materials. In the photograph, the top row displays whole chaga voucher samples (WChV1–WChV6) sourced from various geographical regions, with coarsely ground powders of the same chaga samples shown just below. The middle row depicts *Inonotus obliquus* (*I. obliquus*) mycelium samples grown in liquid culture (IoMyc1 and IoMyc2) and Petri dish (IoMyc3). The middle row also shows common grain substrates (brown rice, oats, and sorghum) used in fermentation. The bottom row shows commercial chaga products.

Whole chaga vouchers (WChV1-6), regardless of origin, appeared as woody chunks with a bright cinnamon-brown interior and a thin outer layer of almost black crust, characterized by a coarse and cracked texture (Figure 1, top row). Samples of *I. obliquus* mycelium (IoMyc) grown in liquid culture (IoMyc1,2) or agar Petri dish (IoMyc3) and freeze-dried ranged in color from sand (IoMyc1 and IoMyc3) to nearly pure white (IoMyc2) (Figure 1, middle row). Cooked, autoclaved, lyophilized grains (brown rice, oats, and sorghum) were cream-colored, with oats presenting a slightly darker hue (Figure 1, middle row, right). Among commercial products, the chaga canker 1:1 extract (ChExt11) was distinctly dark brown, whereas the fermented grain products appeared in various shades of beige and light brown (Figure 1, bottom row).

### 2.2. Polysaccharides in Authentic Chaga Canker and Commercial Products

#### 2.2.1. β-Glucan Quantification

**Figure 2:**
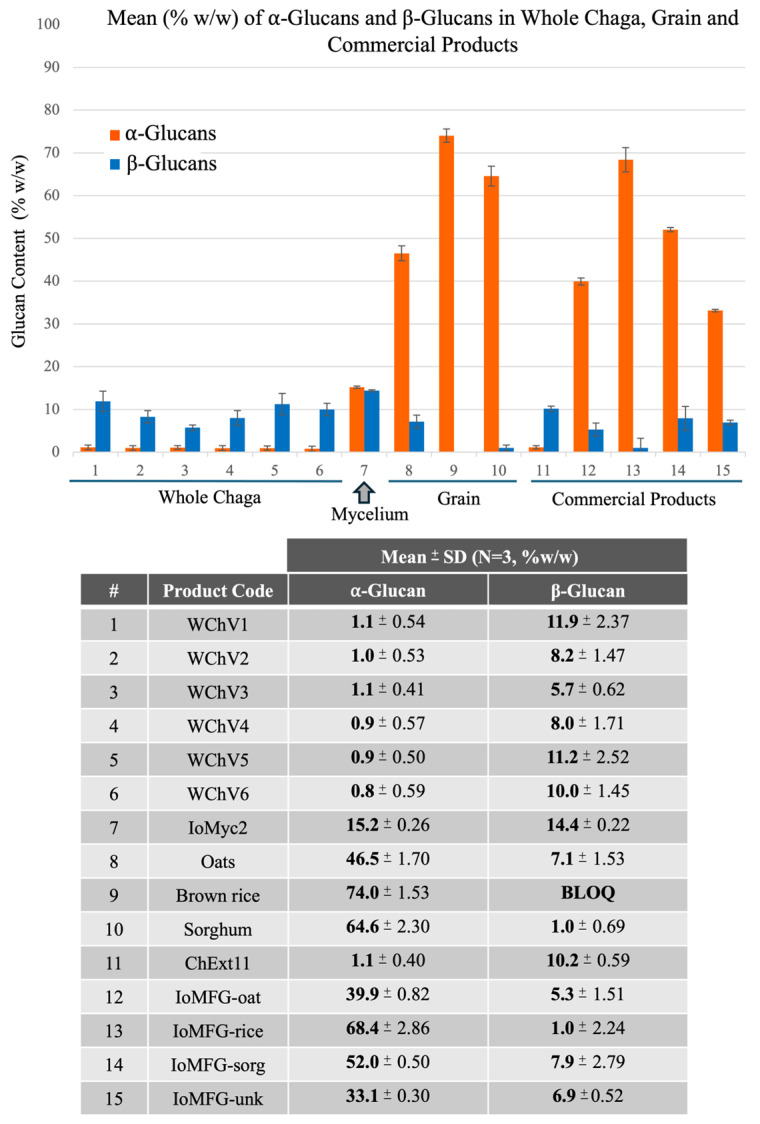
Quantification of α-glucans and β-beta glucans, in chaga, *I. obliquus* mycelium, grain, and commercial products. Glucan quantification was conducted using the enzymatic assay by Megazyme^®^ (Yeast & Fungi kit, K-YBGL). The assay was run in triplicate for each sample, and mean values are represented in the graph, with error bars representing standard deviation. Numbers on the X-axis in the graph correspond to sample numbers in the table below.

Megazyme^®^ Yeast & Mushroom β-Glucan assay (K-YBGL) analysis of β-glucan levels demonstrated that WChV1-6 contained approximately 5.7–11.9% β-glucans, with slightly higher levels in the pure mycelium sample IoMyc2 (14.4%) (Figure 2). On average, β-glucan levels were lower in IoMFG products than in authentic chaga canker. IoMFG-oat and IoMFG-rice showed β-glucan content that was comparable to their cereal substrates, yielding ~1% in rice-based materials and ~5% in oat-based products; only the sorghum-based sample (IoMFG-sorg) had somewhat higher β-glucan content than pure sorghum. Significantly higher levels of α-glucans were found in grain samples (46–74%) as well as fermented grain products (33–68%) compared to authentic chaga canker, which contained less than 2% α-glucans, or pure mycelium, IoMyc2, which had 15.2% α-glucans. ChExt11 displayed α-glucan and β-glucan levels comparable to WChV1-6 (Figure 2).

#### 2.2.2. Starch Detection Using Lugol’s Reagent (I_2_/KI)

**Figure 3:**
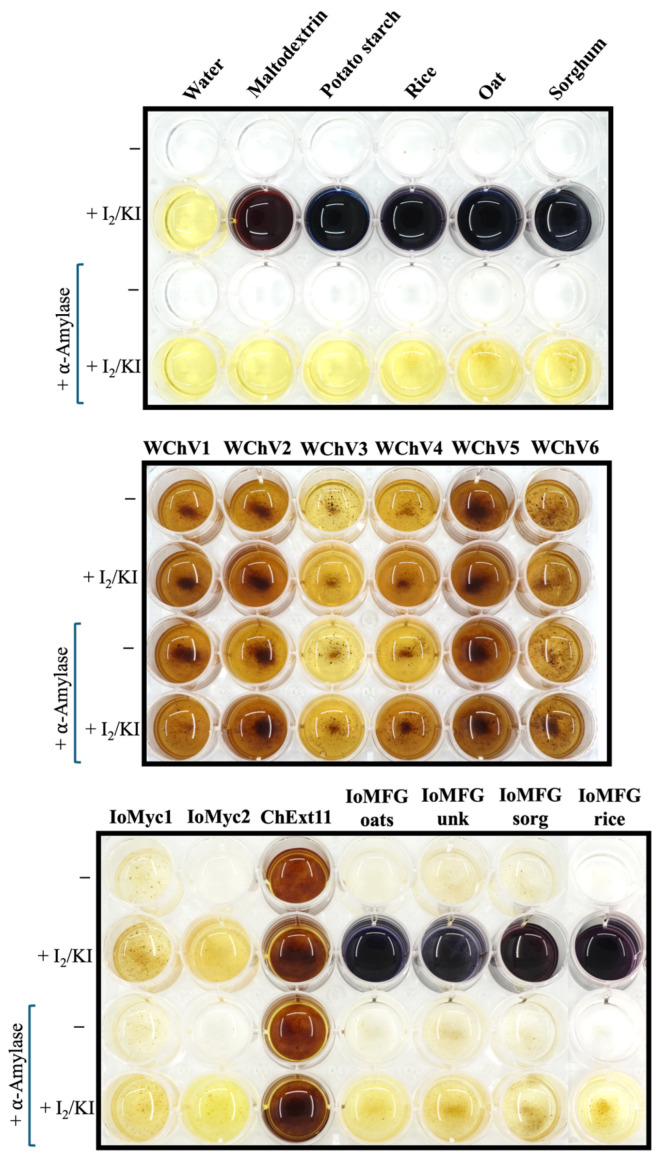
Colorimetric detection of starch in chaga, grain, and commercial products, using Lugol’s (I₂/KI) reagent. In each well, 0.5 mL of suspension containing 5 mg/mL of powdered sample in distilled water. In each panel, the first and third rows labeled (−) depict the negative control wells where no Lugol’s reagent was added, while the second and bottom rows labeled (+I_2_/KI) were colorized with 50 µL of Lugol’s solution. The bottom two rows (+α-Amylase have been treated overnight with ɑ-Amylase (30 U/mL), prior to the addition of Lugol’s reagent to the bottom row.

Starch detection using Lugol’s (I_2_KI) reagent revealed the presence of starch, as well as its degradation product, maltodextrin in fermented grain products, as evident from the development of blue-purple color in these samples, upon addition of Lugol’s reagent (Figure 3). None of the authentic chaga, nor pure *I. obliquus* mycelium samples showed a significant color change upon Lugol’s reagent addition, apart from the yellow color of iodine. After overnight digestion with α-amylase the blue-purple color was no longer detected in either the grain samples or fermented grain products, confirming that this color change was caused by the presence of starch in these samples. All authentic chaga samples (WChV1-6), *I. obliquus* mycelium (IoMyc), and chaga canker 1:1 extract (ChExt11) had the same color before and after α-amylase treatment. These data suggest that, in contrast to authentic chaga, fermented grain products contain a significant amount of starch (Figure 3).

### 2.3. Melanin Evaluation in Chaga Dietary Supplements

The absorbance values from alkaline extracts (Table 1) ranged from 0.4854 (dilution factor 20) in WChV1-6 and up to 0.7685 (dilution factor 10) in ChExt11. These values reflect the absorbance of solubilized compounds extracted with NaOH, a method commonly used to concentrate melanin-like pigments. In contrast, IoMFG and IoMyc1 had trace absorbances, ranging from 0.0313 to 0.0492 (dilution factor 1). 

**Figure 4:**
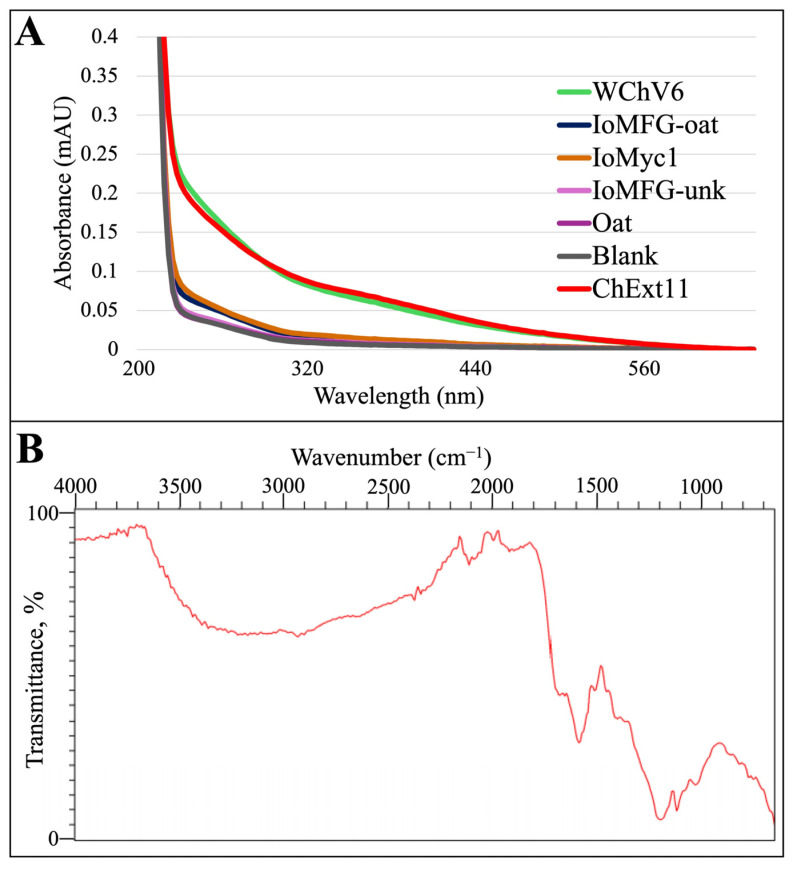
Spectral analysis of the melanin extracts from chaga canker, related product samples, and purified melanin reference. (**A**) UV-Vis absorbance spectra of whole chaga canker (WChV6), chaga canker 1:1 extract (ChExt11), mycelia fermented grain products (IoMFG), *I. obliquus* mycelium (IoMyc1), oat substrate, and a 0.05 M NaOH blank. Samples were chosen to compare melanin-rich (WChV6, ChExt11) and melanin-deficient (IoMFG, IoMyc1, oat) materials, with the NaOH blank serving as a baseline. WChV6 and ChExt11 exhibit a broad melanin-associated absorption profile, while IoMFG, IoMyc1, and the oat substrate show minimal absorbance. (**B**) ATR-FTIR spectrum of the purified melanin reference prepared from ChExt11, demonstrating key functional group absorbances associated with melanin structure, including hydroxyl (3400 cm^−1^), aromatic C=C stretching (1650 cm^−1^), and phenolic C–O stretching (1200–1300 cm^−1^).

UV-Vis spectra of extracted pigments from WChV6 and ChExt11 displayed a characteristic decreasing absorbance from 200 to 600 nm (Figure 4A). In contrast, IoMyc, IoMFG-oat, and the oat substrate had spectra nearly identical to the 0.05 M NaOH blank, with only trace absorbance in the UV and visible light ranges. This aligns with the observation that IoMFG and oat samples yielded white to pale brown material following melanin extraction, indicating minimal pigment presence. Sorghum, rice grains, and their fermented grain products were excluded, as no material was recovered from the extraction procedure.

The ATR-FTIR spectrum of the purified melanin reference (Figure 4B) exhibited broad O–H stretching at approximately 3400 cm^−1^, aromatic C=C stretching near 1650 cm^−1^, and signals in the 1200–1300 cm^−1^ range associated with phenolic C–O stretching, consistent with the functional groups associated with fungal melanin.

### 2.4. HPTLC Identification of Chaga

**Figure 5:**
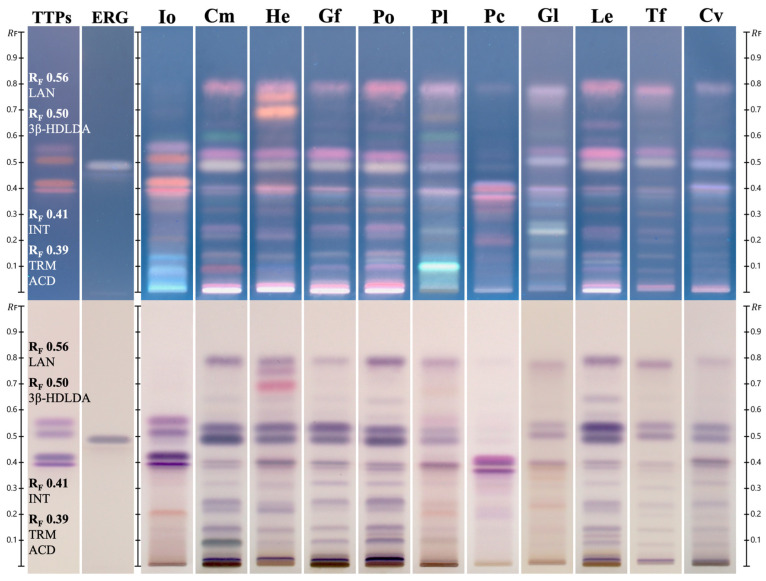
Comparison of the HPTLC chemical fingerprints of whole mushroom, conk, and sclerotium vouchers from 11 fungi species. Top: 366 nm UV visualization. Bottom: white light visualization. Both images were captured following anisaldehyde/sulfuric acid derivatization. The lanes from left to right begin with triterpenoids (TTPs), which are from highest to lowest R_F_ value: lanosterol (LAN; R_F_ 0.56), 3ß-hydroxylanosta-8,24-dien-21-al (3ß-HDLDA; R_F_ 0.50), inotodiol (INT; R_F_ 0.41), and trametenolic acid (TRM ACD; R_F_ 0.39). Chaga canker is the only track with these triterpenoids visibly present. The second lane contains ergosterol (ERG), present in each species. Fungal species are abbreviated as follows: chaga (Io), cordyceps (Cm), lion’s mane (He), maitake (Gf), Oyster (Po), phellinus (Pl), poria (Pc), reishi (Gl), shiitake (Le), tremella (Tf), and turkey tail (Cv).

HPTLC fingerprinting, using an identification method previously developed by Nammex, was performed to compare the chemical profiles of chaga against 10 other fungal species commonly found in dietary supplement products (Figure 5). Visualization after anisaldehyde/sulfuric acid derivatization is shown for 366 nm UV (Figure 5, Top) and white light (Figure 5, Bottom). While ergosterol appears ubiquitously across the species at varying strengths, the triterpenoids lanosterol, 3ß-hydroxylanosta-8,24-dien-21-al, inotodiol, and trametenolic acid are uniquely observed in the chaga canker. In addition to its marker compounds, chaga canker’s unique composition is also reflected by the lack of many of the bands found in the chemical fingerprint of the other species.

**Figure 6:**
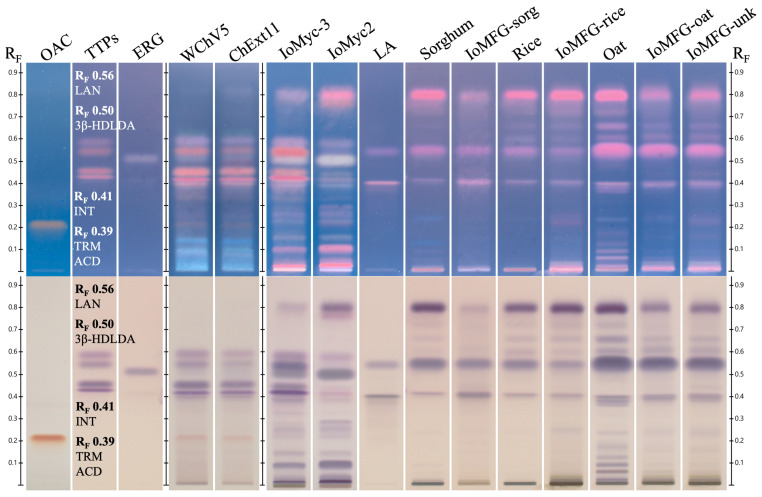
HPTLC chromatograms of chemical markers, whole chaga voucher (WChV5), chaga canker 1:1 extract (ChExt11), *I. obliquus* mycelium grown on an agar plate (IoMyc3), liquid culture *I. obliquus* mycelium (IoMyc2), mycelia fermented grains (IoMFG), and associated grain substrates (sorghum, brown rice, and oat). The first reference lane contains osmundacetone (OAC), observed only in ChExt11 and WChV5. The next track to the right contains triterpenoid (TTPs) standards, with retention factors (R_F_) from high to low belonging to lanosterol (LAN; R_F_ 0.56), 3ß-hydroxylanosta-8,24-dien-21-al (3ß-HDLDA; R_F_ 0.50), inotodiol (INT; R_F_ 0.41), and trametenolic acid (TRM ACD; R_F_ 0.39). The tracks titled ERG and LA contain ergosterol and linoleic acid standards, respectively. ChExt11, WChV5, and IoMyc3 display characteristic triterpenoid bands. IoMyc2 lacks the triterpenoids but has a more intense ergosterol band. IoMFG and grain substrates show a distinct lack of triterpenoid content but an intense linoleic acid band, emphasizing compositional differences.

HPTLC chromatograms revealed distinct triterpenoid markers in WChV5, ChExt11, IoMyc3, aligning with reference standards for inotodiol, trametenolic acid, lanosterol, and 3β-hydroxylanosta-8,24-dien-21-al (Figure 6). After derivatization with anisaldehyde/sulfuric acid, these triterpenoids were readily visualized. By contrast, IoMyc1 and IoMFG products lacked these key triterpenoid bands (Figure 6). Betulin and betulinic acid coeluted and were not detected in any of the samples.

**Figure 7:**
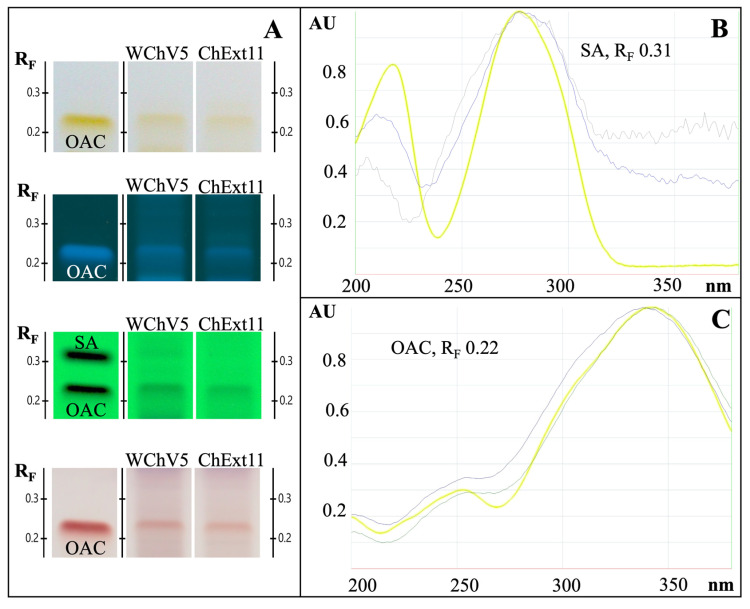
Chaga canker (WChV5) and chaga canker 1:1 extract (ChExt11) phenolic HPTLC chromatograms and UV-Vis absorbance spectra. (**A**) HPTLC bands corresponding to the standards for osmundacetone (OAC) and syringic acid (SA) are compared against WChV5 and ChExt11, with the images from top to bottom being captured under white light, 366 nm UV, and 254 nm UV after development, and then white light after derivatization. HPTLC-densitometer absorbance spectra captured after the development of syringic acid (SA; (**B**)) and osmundacetone (OAC; (**C**)). Spectra of these standards are highlighted yellow, with the bands of corresponding R_F_ positions in WChV5 and ChExt11 overlaid.

The methanol extract of WChV5 consistently displayed bands corresponding to osmundacetone (R_F_ 0.22) and syringic acid (R_F_ 0.31) visible under 254 nm UV, with osmundacetone also appearing as a yellow band under white light, 366 nm UV light, and white light following derivatization with anisaldehyde/sulfuric acid (Figure 7A). UV-Vis absorbance spectra confirmed the presence of syringic acid and osmundacetone through comparison to reference standards of these compounds (Figure 7B,C).

### 2.5. LC-ELSD/DAD/PDA Detection of Chaga Chemical Constituents

**Figure 8:**
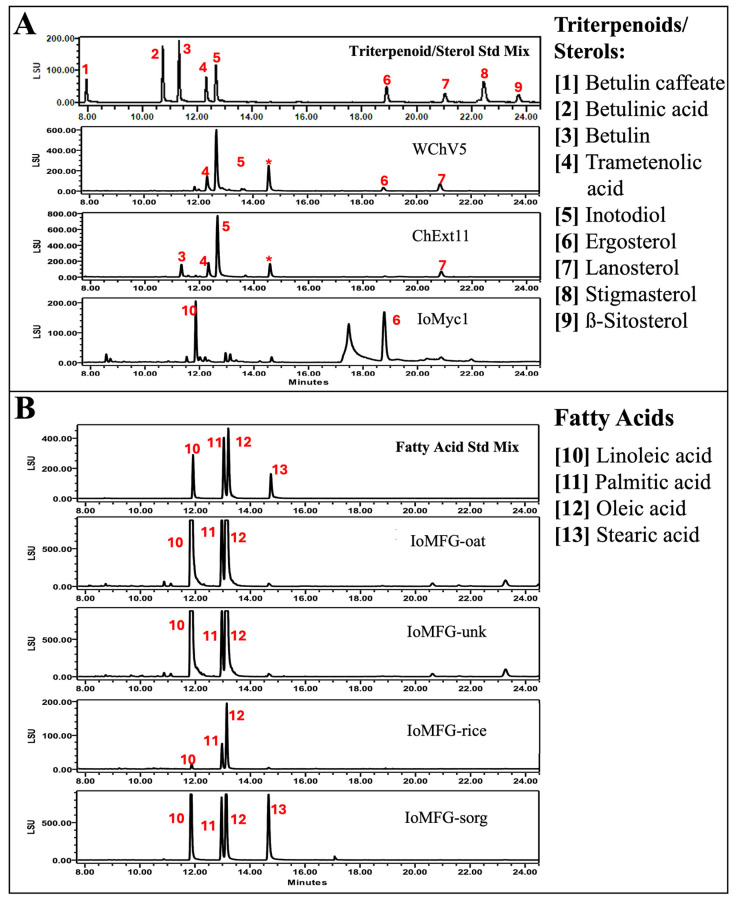
LC-ELSD chromatograms of triterpenoid and fatty acid standards compared with whole chaga canker (WChV5), chaga canker 1:1 extract (ChExt11), *I. obliquus* mycelium (IoMyc1), and mycelia fermented grains (IoMFG). (**A**) Chromatograms of a nine-compound triterpenoid/sterol standard mix, with key peaks labeled. Whole chaga and chaga canker 1:1 extract displays prominent peaks for trametenolic acid (4), inotodiol (5), lanosterol (7), aligning with authentic chaga composition. In contrast, IoMyc1 exhibits ergosterol (6) as its primary sterol. The peak labeled with an asterisk (*) was initially considered unknown but was later likely identified as 3β-hydroxylanosta-8,24-dien-21-al based on HPTLC and HPLC-DAD analyses. (**B**) Chromatograms of fatty acid standard mix. IoMFG products lack characteristic chaga triterpenoids and instead show dominant peaks for fatty acids, including linoleic (10), palmitic (11), oleic (12), and stearic (13) acids, reflecting their grain substrate composition.

LC-ELSD chromatograms of WChV2, WChV5, and ChExt11 displayed prominent peaks corresponding to triterpenoids, including inotodiol, trametenolic acid, and lanosterol (Figure 8A). In contrast, IoMyc1 and IoMFG lacked these triterpenoids entirely. IoMFG products exhibited intense peaks for fatty acids, including linoleic, palmitic, oleic, and stearic acids, which were absent or present only in trace amounts in authentic whole chaga canker (Figure 8B). IoMyc1 displayed a peak corresponding to linoleic acid.

**Figure 9:**
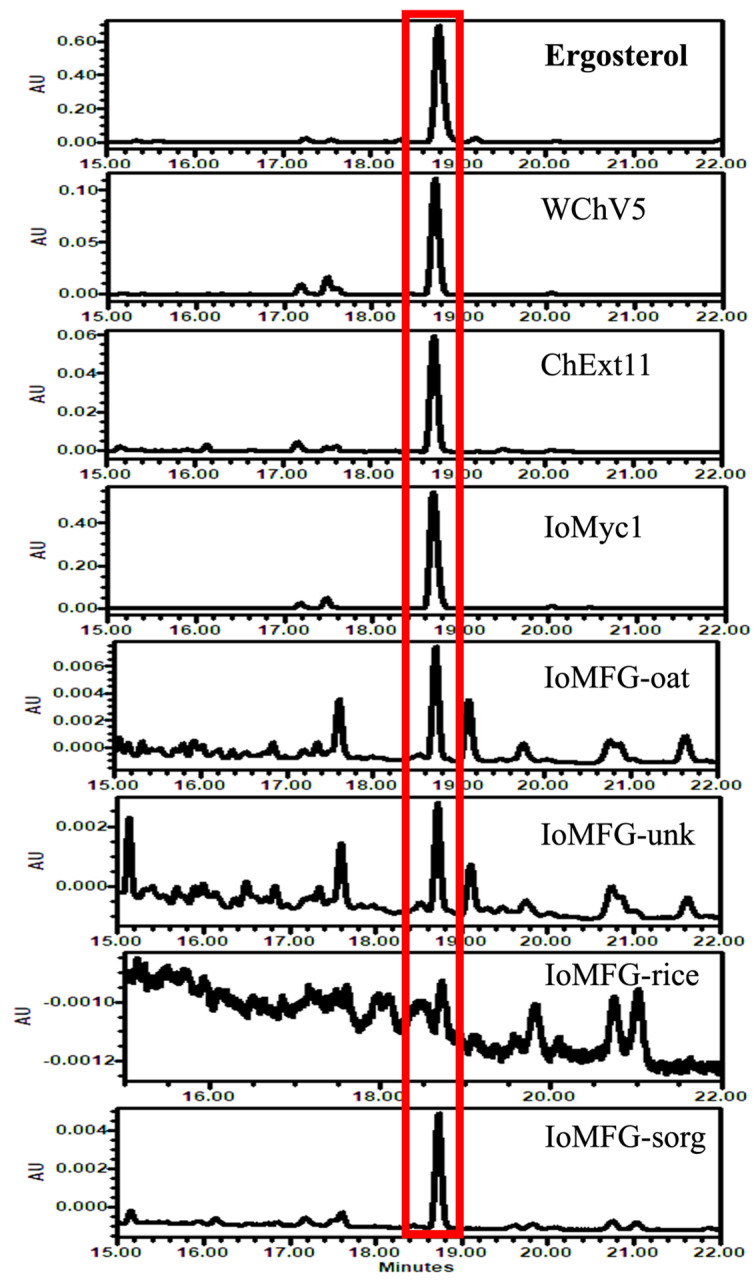
LC-UV 281 nm chromatograms comparing ergosterol contents in whole chaga canker (WChV5), chaga canker 1:1 extract (ChExt11), *I. obliquus* mycelium (IoMyc1), and mycelia fermented grain (IoMFG) products. WChV5 and ChExt11 exhibit low ergosterol levels, while IoMyc1 shows a strong ergosterol peak, confirming its fungal origin. IoMFG products contain detectable but significantly lower ergosterol levels, approximately one-tenth the peak height of that found in WChV5.

LC-PDA analysis at 281 nm (Figure 9) assessed ergosterol content, with greater peak intensities observed in *I. obliquus* mycelium (IoMyc1) than those observed in WChV5 and ChExt11. IoMFG showed the lowest ergosterol signals, with peak heights approximately one-tenth of those in WChV5.

**Figure 10:**
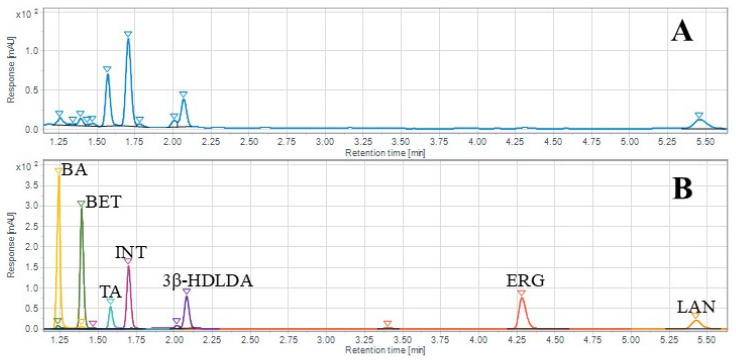
HPLC-DAD chromatograms of (**A**) whole chaga canker (WChV5) and (**B**) chemical reference standards for betulinic acid (BA), betulin (BET), tramentenolic acid (TA), inotodiol (INT), 3β-hydroxylanosta-8,24-dien-21-al (3 β -HDLDA), ergosterol (ERG), and lanosterol (LAN).

Table 2 provides a summary of triterpenoid contents from WChV1-6 and ChExt11. The most abundant triterpenoid was inotodiol (0.19%), followed by trametenolic acid (0.09%), 3β-hydroxylanosta-8,24-dien-21-al (0.09%), and lanosterol (0.06%). None of the triterpenoids were detected in either the liquid cultured *I. obliquus* mycelium or fermented grain samples. Statistical analysis is also reported for each triterpenoid, including limit of detection (LOD), limit of quantification (LOQ), and correlation coefficient (R^2^). Across the triterpenoids, the LOD and LOQ ranged from 0.86 to 4.96 ng and 2.61 to 15.02 ng, respectively. Correlation coefficients ranged from 0.9998 to 1.0000, indicating each triterpenoid calibration curve was well fit by a linear regression model. Figure 10 shows the HPLC-DAD chromatograms of WChV5 compared to triterpenoid and ergosterol reference standards.

### 2.6. ^1^H NMR Spectral Fingerprinting of Chaga and Commercial Products

**Figure 11:**
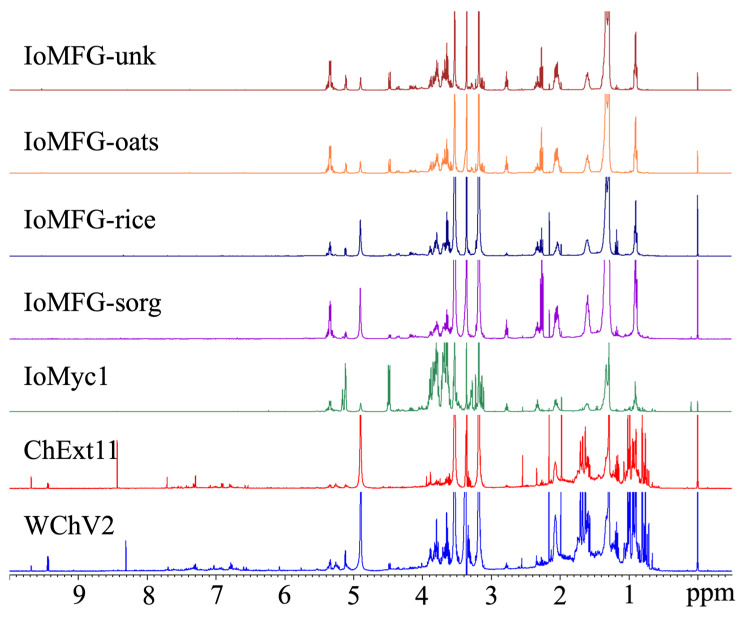
^1^H NMR spectra of whole chaga (WChV2), chaga canker 1:1 extract (ChExt11), *I. obliquus* mycelium (IoMyc1), and mycelia fermented grain (IoMFG) products. Both WChV2 and ChExt11 exhibit distinct signals in the 6.5–9 ppm region, absent in IoMyc1 and IoMFG products. Additional spectral differences between chaga canker-based products and fermented grain products are evident in the 0.5–2.5 ppm and 6.5–9 ppm regions, corresponding to compositional variations.

**Figure 12:**
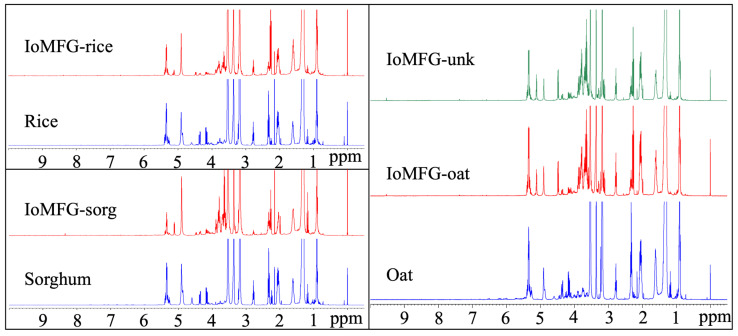
^1^H NMR spectra of mycelia fermented grain products (IoMFG) compared to their respective cooked, autoclaved, and freeze-dried grain substrates (rice, sorghum, and oat). In each case, the spectral pattern of the fermented grain closely resembles that of its substrate, indicating a predominantly unchanged composition. This suggests that fungal metabolism during fermentation does not significantly alter the grain’s chemical profile, with the major detected components likely originating from the substrate rather than fungal metabolites.

¹H NMR spectral fingerprinting revealed distinct compositional differences between WChV2, ChExt11, IoMyc1, and IoMFG products (Figure 11). WChV2 and ChExt11 displayed peaks in the aliphatic (0.5–2.5 ppm) and aromatic regions (6.0–9.0 ppm), with IoMyc and IoMFG showing lower to no signal in these regions. The sugar region (3.0–4.0 ppm) shows overlapping signals attributable to polysaccharides. Comparisons of IoMFG to their corresponding grains (rice, oat, and sorghum) showed near-superimposable spectra (Figure 12).

### 2.7. LC-QToF-MS Metabolomic Analysis of Chaga Dietary Supplements

**Figure 13:**
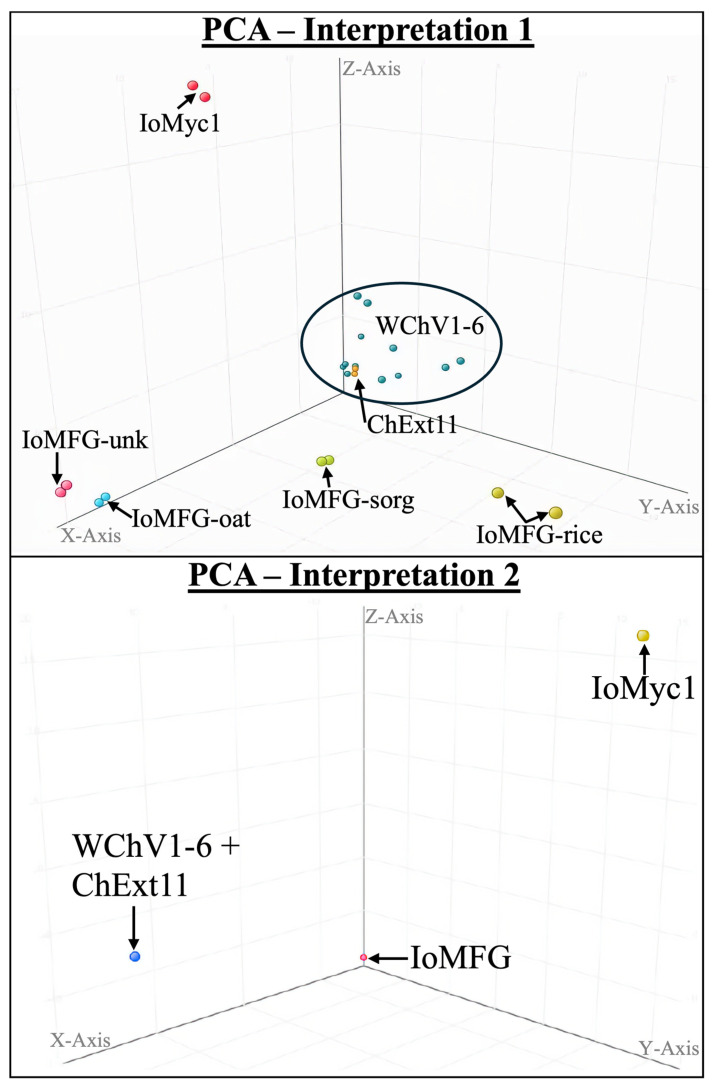
Principal component analysis (PCA) of LC-QToF-MS metabolomic data comparing whole chaga canker (WChV1-6), chaga canker 1:1 extract (ChExt11), *I. obliquus* mycelium (IoMyc1), and mycelia fermented grain products (IoMFG). (Top) PCA score plot with axes representing variance explained by the first three principal components: Component 1 (X-axis, 47.78%), Component 2 (Y-axis, 14.89%), and Component 3 (Z-axis, 8.36%). The close clustering of WChV1–6 and ChExt11 indicates high metabolic similarity, whereas IoMFG forms separate clusters, reflecting distinct compositional differences. IoMyc1 also clusters separately, demonstrating its unique metabolic profile. (Bottom) Alternative PCA interpretation with Component 1 (X-axis, 57.72%), Component 2 (Y-axis, 42.28%), and Component 3 (Z-axis, 42.28%), further illustrates the compositional divergence of fermented grain products and *I. obliquus* mycelium from authentic chaga canker.

The LC-QToF-MS metabolomic analysis revealed distinct chemical profiles among WChV1–6, ChExt11, IoMyc1, and IoMFG products. PCA for Interpretation 1 demonstrated tight clustering of WChV1–6 and ChExt11 (Figure 13, Top). In contrast, IoMFG products formed dispersed clusters, away from both chaga canker materials and IoMyc, reflecting minimal fungal modification. Interpretation 2 PCA further segregated chaga canker, IoMyc, and IoMFG into distinct groups (Figure 13, Bottom).

**Figure 14:**
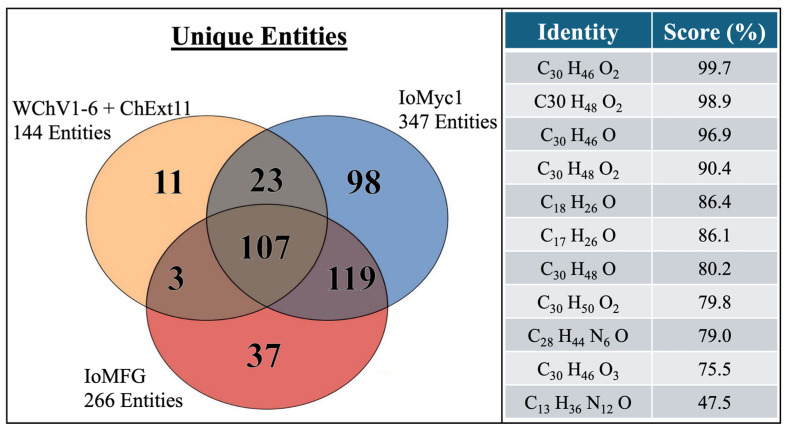
The entities from LC-QToF-MS that are exclusive or shared among whole chaga canker (WChV1-6), *I. obliquus* mycelium (IoMyc1), and mycelia fermented grains (IoMFG). (Left) Venn diagram showing unique molecular entities identified in WChV1-6, ChExt11, IoMyc1, and IoMFG. (Right) Table summary of molecular formulas and confidence scores (%) for unique entities in WChV1–6 and ChExt11, identified through untargeted LC-QToF-MS metabolomic profiling, PCA classification, and algorithmic formula generation. The detected compounds align with triterpenoids and sterol derivatives, reinforcing their role as key chemical markers distinguishing authentic chaga canker.

Venn diagram analysis (Figure 14, Left) identified 11 unique molecular entities exclusive to WChV1–6 and ChExt11, including compounds with molecular formulas (Figure 13, Right). Statistical filtering (ANOVA, *p* < 0.05; fold change > 2.0) confirmed their significance as chaga-specific biomarkers.

## 3. Discussion

### 3.1. Analytical Methods and Their Limitations

Multiple analytical methods were used to authenticate chaga (*I. obliquus*) products and distinguish them from fermented grain alternatives, including Lugol’s reagent for starch detection, Megazyme^®^ β-glucan (K-YBGL), HPTLC, LC-ELSD, LC-UV, melanin-focused UV-Vis/FTIR analyses, ^1^H NMR, and LC-QToF-MS. Each method, however, has inherent constraints: Lugol’s reagent provides primarily qualitative starch detection; the Megazyme^®^ β-glucan assay (K-YBGL) is an accurate method for measuring fungal β-(1,3)–(1,6) glucans in mushrooms and yeast but, as the manufacturer explicitly warns, products containing grain will elevate results due to detection of cereal β-glucans and cellulose; HPTLC requires visual interpretation of semiquantitative banding patterns; LC-ELSD and LC-UV can be limited by overlapping peaks, non-UV absorbing compounds, or low abundance constituents; and melanin assessments via UV-Vis and FTIR may be influenced by other chromophores and do not fully confirm pigment identity without orthogonal techniques. Additionally, while ^1^H NMR yields a comprehensive metabolomic fingerprint, it may miss low-concentration compounds and, like LC-QToF-MS, requires extensive instrumentation and expertise. Despite these individual limitations, using multiple complementary assays together provides a robust framework for product authentication, as the strengths of each method offset the weaknesses of the others.

### 3.2. Physical Appearance Differences Between Whole Chaga Canker and Fermented Grain Products

Physical appearance offers a distinction between authentic chaga and its extracts with fermented grain products (Figure 1). WChV1–6 consistently appears as woody chunks with a dark, crusted outer layer and cinnamon brown interior, while IoMFG products show lighter, beige-brown coloration depending on the cereal substrate. The visual cues observed in whole chaga samples align with previous descriptions of *I. obliquus* canker morphology [2,3]. The distinct coloration and textural differences observed among whole chaga canker, cultured mycelium, and commercial products suggest substantial compositional differences, notably in their pigment contents. Pure mycelium was pale in color and would be difficult to distinguish from finely ground grain based on physical appearance alone, but our subsequent chemical analyses highlight important distinctions between these samples.

### 3.3. Polysaccharide Composition as a Key Differentiator

The α-glucan, and β-glucan data shown in Figure 2 confirm fundamental polysaccharide distinctions between whole chaga canker and fermented grains. WChV1–6 contain low amounts of α-glucans (0.8–1.1%), while β-glucans ranged from 5.7–11.9%, a modest amount compared to other mushroom species [49,50] but characteristic of authentic chaga [18,51,52]. ChExt11 showed a similar pattern, with low α-glucans (1.1%), and β-glucans at 10.2%, reinforcing its authenticity as a genuine chaga canker product. In contrast, pure grain and fermented grain “chaga” formulations contained high α-glucans (33.1–74.0%). 

Lugol’s reagent effectively differentiates fermented grain products from whole chaga canker. Upon addition of Lugol’s reagent, IoMFG products turned navy blue to purple, indicating the presence of starch and maltodextrin, both absent in WChV1–6 (Figure 3). This colorimetric response aligns with the known high starch content of grains [45,46,47]. After overnight digestion with α-amylase, the blue-purple color was no longer observed in grain and mycelia fermented grain samples, confirming the color change observed prior to α-amylase treatment was starch-driven. Some sources note that Lugol’s reagent can produce a dark brown color in the presence of glycogen, a polysaccharide found in mushrooms with α-(1,4) and (1,6) glucose linkages, like starch [53,54,55]. However, no significant color change was observed in WChV1–6 or IoMyc. The purple hue observed in IoMFG products was reproducible by mixing starch and maltodextrin together with Lugol’s reagent, further supporting maltodextrin’s role in the reaction. The presence of starch in grain-based products aligns with their high α-glucan content, indicating that starch is the main contributor to the high α-glucan levels in these products. Notably, liquid culture *I. obliquus* mycelium (IoMyc2), despite containing 15.2% α-glucans, did not show a significant color change upon addition of Lugol’s reagent, confirming that the mycelium itself does not contain starch and does not contribute to the starch positive result observed in fermented grains. These findings demonstrate starch as a reliable marker for detecting grain-derived material, particularly in dietary supplements that may not clearly label grain as an ingredient.

The α- and β-polysaccharide content of IoMyc2 differed from that of WChV1–6, with higher levels of α-glucan (15.2%) and β-glucan (14.4%). These differences likely reflect metabolic adaptations to nutrient-rich growth conditions, where the mycelium prioritizes glycogen-like α-glucan storage over structural β-glucan synthesis. This is supported by previous findings, where submerged fermentation *I. obliquus* mycelium produced an anomeric NMR signal ratio of 1.7:1 glycogen-like α-glucan to β-glucan, in addition to IR signals attributable to α-glycosidic linkages (830 cm^−1^) in the mycelium but not chaga canker polysaccharide fractions [44]. The research also found that submerged fermentation mycelium contained higher molecular weight polysaccharides than chaga canker, consistent with the production of storage rather than structural polysaccharides [44]. Lugol’s reagent, which normally stains helical α-glucans like glycogen dark brown, showed no reaction to liquid-cultured *I. obliquus* mycelium in our study (Figure 3). This suggests that the α-glucans in our mycelium samples either lack the helical structure necessary to produce color change in addition to Lugol’s reagent or that compounds of this nature are present at levels below detection in these samples. Further structural analysis is needed to clarify its branching, chain length, and linkage specificity.

β-Glucan analysis effectively differentiates authentic chaga canker from grain-derived material (Figure 2). Our results are consistent with previous research reporting that authentic chaga canker contains approximately 8–15% β-glucans [18,51,52]. While IoMFG products contained some β-glucans (1.0–6.9%), values were consistently lower than in WChV1–6, and α-glucan levels were disproportionately high (33.1–68.4%). IoMFG β-glucans are likely attributable to the detection of cereal β-glucans and cellulose, aligning closely with reported concentrations found in oats, brown rice, and sorghum [45,46,47]. This suggests that fermented grain product polysaccharides likely originate from the cereal substrate, rather than fungal biomass.

The high α-glucan levels in mycelia-fermented grain products contrast sharply with the low α-glucan levels in authentic chaga. These results highlight the importance of measuring multiple polysaccharide markers (α- and β-glucans, and starch) for accurate chaga canker authentication. The α-glucan to β-glucan ratio may serve as a useful metric for assessing product composition and ensuring supplement quality.

### 3.4. Melanin as a Marker for Chaga Identification

Previous work demonstrated that alkaline extract from the chaga canker, as well as mycelium grown in submerged fermentation, displayed a UV-Vis absorbance spectrum characteristic of melanin [1,41,48]. *I. obliquus* melanins were subsequently characterized as allomelanin in the chaga canker and eumelanin in mycelium: despite their structural differences, these compounds share a similar UV-vis spectrum. The pronounced UV-Vis absorbance and ATR-FTIR spectral features in WChV1–6 and ChExt11 samples confirm melanin as a definitive chemical marker for authentic chaga canker. Melanin’s broad absorption profile in the UV-Vis spectrum (Figure 4A) and its characteristic IR signatures align with prior studies on fungal melanin, which emphasize its polyphenolic and macromolecular structure [4,48,56]. The high absorbance values in WChV1–6 and ChExt11 correlate with its dark pigmentation (Figure 1). Conversely, the negligible absorbance from IoMFG products reflects their lack of melanin. Although there are reports in the literature of melanin production in *I. obliquus* mycelium grown in submerged cultivation [41], we did not find any evidence of melanin in IoMyc1. This is likely due to differences in the cultivation conditions and media used in our study, which can have a profound influence on the production of secondary metabolites [42]. This study did not endeavor to produce mycelium with a specific biochemical composition but rather to produce pure mycelium as a control for comparison to mycelia fermented grain products.

### 3.5. Triterpenoid and Sterol Profiles Distinguish Chaga Canker from Mycelia Fermented Grain

The distinct triterpenoid profiles determined by LC-ELSD in this study reflect the metabolic specialization of whole chaga canker in contrast to *I. obliquus* mycelium and mycelia fermented grain products (Figure 5 and Figure 8). The detection of inotodiol, trametenolic acid, and lanosterol in chaga is consistent with previous reports identifying these compounds as chaga canker-specific metabolites [34,37,42,57]. LC-DAD quantification confirmed that wild chaga canker contained inotodiol at 0.19% *w*/*w* on average, successfully exceeding the previously recommended threshold of 0.1165% *w*/*w* for chaga authentication [37]. The measured content of 3β-hydroxylanosta-8,24-dien-21-al (0.09%) was notably lower than the previously reported 0.1717% *w*/*w*. However, our findings suggest that the 0.1717% value may be overestimated, potentially due to differences in extraction methodology, quantification techniques, or sample selection criteria. This absence of key bioactive triterpenoids in IoMFG products reinforces that fungal fermentation on grain substrates fails to replicate the chemical complexity of whole chaga canker, consistent with previous research [42,58]. Interestingly, lanosterol and ergosterol were detected in pure mycelium samples IoMyc1, IoMyc2, and IoMyc3, and triterpenoids in IoMyc3 (Figure 6 and Figure 8). Both IoMyc1 and IoMyc2 were grown in liquid culture; IoMyc1 was grown in a simple medium (yeast extract, dextrose, malt), and IoMyc2 was grown in buffered peptone broth containing amino acids. The use of buffered peptone promoted faster growth and increased secondary metabolite production, aligning with previous studies, focused on optimizing submerged cultivation of *I. obliquus* mycelium for enhanced metabolite yield [42]. Notably, high triterpenoid levels were found in *I. obliquus* mycelium grown on a Petri dish, suggesting its capacity to produce these metabolites under certain conditions. The total absence of these triterpenoids in fermented grain products emphasizes the fundamental biochemical differences between these products and pure mycelium.

Whole genome analysis of *I. obliquus* suggests that this organism possesses the biosynthetic enzymes necessary to produce betulin and betulinic acid [59]. However, we found these triterpenoids present only in trace amounts or entirely absent in WChV1–6, as well as IoMyc and IoMFG products. This suggests that betulin and betulinic acid are not reliable markers for chaga, despite previous reports of high betulin content in chaga from certain geographic areas [27,49].

LC-PDA detection of ergosterol further differentiates *I. obliquus* mycelium and chaga canker from fermented grain (Figure 9). While ergosterol is a fungal membrane sterol, its low levels in WChV1–6 contrast sharply with its abundance in IoMyc1. The minimal detection of ergosterol in IoMFG reflects dilution by grain substrates. While ergosterol alone cannot authenticate chaga canker, its detection in chaga products provides a proxy for fungal biomass, aiding quality control in mixed matrices [60].

### 3.6. Phenolic and Fatty Acid Profiles Highlight Additional Compositional Differences

HPTLC phenolic profiling (Figure 6 and Figure 7) identified osmundacetone and syringic acid, both reported as chaga-associated phenolics, exclusively in the whole chaga canker [16,20,61,62,63,64,65]. This suggests that *I. obliquus* mycelium and fermented grains not only lack key triterpenoids, but do not replicate the polyphenolic profile of chaga canker, further differentiating them from the authentic material.

The fatty acid profiles of IoMFG products, dominated by linoleic and oleic acids, mirror lipid patterns in cereal grains, confirming their substrate-based composition. These results are in alignment with reports of fatty acids in *I. obliquus* fermented oat products [58]. The absence of these lipids in WChV1–6 highlights another layer of compositional divergence, critical for distinguishing fermented grain products in commercial markets.

### 3.7. Metabolomic Analysis Confirms Authenticity Gaps

The HPTLC identification method effectively differentiates authentic chaga canker from other fungal species commonly found in dietary supplements (Figure 5). Among these species, ergosterol was the only shared compound across all samples, whereas chaga’s triterpenoid profile distinguished it from the other species.

Comprehensive HPTLC profiling of triterpenoids (Figure 6) revealed that WChV5, ChExt11, and *I. obliquus* mycelium grown on an agar plate (IoMyc3) exhibited distinct bands corresponding to lanosterol, 3β-hydroxylanosta-8,24-dien-21-al, inotodiol, and trametenolic acid. Osmundacetone was only observed in WChV5 and ChExt11. By contrast, IoMFG products contained no triterpenoids or ergosterol, but showed high linoleic acid (R_F_ 0.26) and triglyceride (R_F_ 0.86) content, closely resembling their grain substrates. 

Beyond individual marker compounds, metabolomic fingerprinting via ¹H NMR and LC-QToF-MS provided a holistic differentiation between chaga canker and mycelia fermented grain products. The ¹H NMR spectra (Figure 11) confirmed that WChV2 and ChExt11 possess distinct aromatic signals (6.0–9.0 ppm), previously attributed to phenolic compounds associated with chaga’s bioactivity [48,66]. Upfield aliphatic regions (0.5–1.5 ppm) displayed stronger and more diverse signals in WChV2, likely reflecting triterpenoid content. The spectral resemblance of IoMFG products to grain substrates (Figure 12) further supports that fungal fermentation under these conditions does not significantly alter grain composition, suggesting limited enzymatic hydrolysis or incorporation of fungal metabolites. This contrasts with commercial claims of “myceliated” grains as bioactive equivalents to chaga, demonstrating potential discrepancies in product composition.

^1^H NMR spectra of IoMFG and grains (Figure 12) exhibit signals at 5.3–5.5 ppm, 2.7–2.8 ppm, and 1.2–1.4 ppm, which correspond to the chemical shifts of linoleic acid [67]. This suggests that unsaturated fatty acids, which HPTLC (Figure 6) and LC-ELSD (Figure 8) also show as predominantly linoleic acid, are a major component of their lipid profile. This aligns with previous findings that *I. obliquus* cultivated on oats retains substantial fatty acid content, unlike chaga canker [58].

The LC-QToF-MS metabolomic data corroborate the chemical uniqueness of WChV1–6 compared to IoMyc1 and IoMFG products. PCA demonstrates the close clustering of WChV1–6 and ChExt11, suggesting that the extract production process preserves the composition of the chaga canker starting material. In contrast, the greater dispersion observed among IoMFG products suggests that fungal fermentation under these conditions does not substantially alter the chemical profile of the grain substrate, reinforcing findings from HPTLC and NMR analyses. Additionally, the close proximity of IoMFG-unk to IoMFG-oat in PCA suggests that the unspecified grain substrate is likely oats. This LC-MS work is consistent with previous research, which used GC-MS to differentiate chaga lipid profiles from mycelial analogs [58], and LC-MS metabolomics as a robust tool for multiple fungal species discrimination [68].

Metabolomic fingerprinting identified 11 entities exclusive to chaga canker materials, with molecular formulas (Figure 14) matching known triterpenoids and sterols (C_30_H_46_O_2_, C_17_H_26_O). These findings align with HPTLC and LC-ELSD data, as well as previous LC-QToF MS analysis of chaga secondary metabolites, which confirm triterpenoids and sterols as distinguishing chemical markers of chaga canker [42]. 

Overall, these NMR, HPTLC, and LC-MS data reinforce the conclusions that authentic whole chaga canker (including its extracts) features a unique metabolic signature, whereas fermented grain products largely mirror the compositional characteristics of their cereal substrates.

## 4. Materials and Methods

### 4.1. Origin of Whole Dried Chaga, Chaga Canker 1:1 Extract, Mycelia Fermented Grain, and Grain Substrate Reference Materials

Vouchers of authentic whole chaga harvested from birch trees were obtained from several geographical locations, including Heilongjiang, China (Nammex, WChV5/6), Alaska, USA (WChV1), Quebec, and Ontario, Canada (WChV2, WChV4), and Finland (WChV3). Pure *I. obliquus* mycelium (IoMyc) was sourced from North Spore culture bank (IO1, lot# 081623-2), and propagated on Petri dishes (10 g/L dextrose, 10 g/L malt extract, 5 g/L yeast extract, 20 g/L agar), and used to inoculate liquid cultures. Pure mycelium samples were produced by Nammex in liquid culture fermentation: IoMyc1 was grown in simple malt and sugar medium (10 g/L dextrose, 10 g/L malt extract, 5 g/L yeast extract), while IoMyc2 was grown in buffered peptone broth (10 g/L dextrose, 20 g/L malt extract, 0.25 g/L yeast extract, 5 g/L buffered peptone 3M^TM^). Mycelium was grown for 3 weeks in 1 L flasks, on a shaker (150 rpm), at 25 °C, filtered and rinsed three times in distilled water, then freeze-dried. Mycelia fermented grain samples were purchased from the internet. Whole oats, short grain brown rice, and sorghum grains were used as substrate reference materials and were prepared by cooking, straining, autoclaving, and freeze drying. Whole chaga canker, whole grains, and pure *I. obliquus* mycelium were ground to a fine powder using a mortar and pestle, while chaga canker 1:1 extract and fermented grain products were already powdered. 

Early work analyzing biochemical profiles of the six whole chaga vouchers demonstrated comparable results (as exemplified in Figure 13). For this reason, in some assays only one representative voucher was used.

### 4.2. Starch Detection with Lugol’s Reagent (I_2_/KI)

For each sample, 50 mg of dry powder was transferred to a glass tube with a screw cap and suspended in 10 mL of distilled water for a final concentration of 5 mg/mL. The suspension was vortexed, and glass tubes were incubated in a hot water bath ~60 °C, for 20 min. The tubes were removed from the water bath and allowed to cool at room temperature to ~40 °C. A 990 µL aliquot of each sample was transferred to a new capped glass tube, to which 10 µL of α-amylase (Megazyme^®^, Bray, Wicklow County, Ireland, Cat Number: E-BSTAA) was added, to a final concentration of 30 U/mL. All samples (with and without α-amylase) were incubated at ~40 °C overnight (>12 h). The following day, 500 µL of each sample was transferred to a 24-well plate, and 50 µL Lugol’s solution (I_2_/KI, Sigma Aldrich, St. Louis, MO, USA, Cat Number: 62650) was added to each well, excluding negative controls. 

### 4.3. Polysaccharide Analysis: α- and β-Glucan Quantification

Quantification of β-Glucans in chaga and commercial products was conducted using the Megazyme^®^ Yeast and Mushroom β-Glucan Assay (K-YBGL ver 08/23), according to the manufacturer’s protocol [69]. Whole chaga canker and chaga canker 1:1 extract samples were prepared following the assay protocol for materials with <10% α-glucan content. Mycelia fermented grain products were prepared using the protocol for samples with >10% α-glucan content, accounting for their high starch-derived α-glucan content. 

### 4.4. Melanin Extraction and Spectral Characterization

#### 4.4.1. Melanin Extraction and Purification

Due to the lack of a commercially available chaga melanin standard, a purified melanin reference was isolated from ChExt11 using an acid-base purification method described previously [70]. Initial extraction began with boiling chaga in water, followed by alkaline hydrolysis in 1 M NaOH and autoclaving to solubilize melanin. Acid precipitation at pH 1 using HCl produced a crude melanin pellet. To enhance purity and solubility, the precipitate underwent two additional purification cycles consisting of dissolution in 1 M NaOH, boiling (100 °C, 20 min), centrifugation (5250× *g*, 1 h), and reprecipitation with HCl. Residual impurities were removed via repeated aqueous washes, and the final melanin was oven dried (80 °C) to constant mass.

For UV-Vis analysis of all samples, a simplified protocol was employed. Test sample powder (5 mg) was suspended in 10 mL of 1 M NaOH, heated in a heating block (110 °C, 20 min), and centrifuged (5250× *g*, 5 min) to isolate solubilized melanin. The supernatants containing the solubilized melanin were analyzed by UV-Vis spectrophotometry. Supernatants were analyzed by UV-Vis spectrophotometry with 2-fold dilution (as necessary to remain in a measurable range for comparative analysis), and all samples were measured with a 2-fold dilution in water for full absorbance spectrum acquisition.

#### 4.4.2. Melanin Spectral Analysis

Absorbance measurements of melanin extracts were recorded at 500 nm using a VWR UV-3100PC spectrophotometer. Additionally, full absorbance spectra (200–600 nm) were recorded using the diode array detector (DAD) of an Agilent 1290 system, operated without a column and using water as the mobile phase.

The purified melanin reference was ground to a fine powder with a mortar and pestle, followed by analysis with an Agilent Cary 630 FTIR equipped with the Diamond-ATR module.

### 4.5. HPTLC Identification and Fingerprinting

#### 4.5.1. Chemicals and Reagents

ACS-grade solvents and reagents, including toluene, methanol, acetic acid, *p*-anisaldehyde, and sulfuric acid, were obtained from Sigma Aldrich.

The following chemicals (purity ≥ 98%) were ordered from ChemFaces (Wuhan, Hubei, China): inotodiol (Lot: CFS202301), trametenolic acid (Lot: CFS202301), betulin (Lot: CFS202101), betulinic acid (Lot: CFS202102), 3ß-hydroxylanosta-8, 24-dien-21-al (Lot: CF202301), osmundacetone (Lot: CFS202401), and syringic acid (Lot: CFS202402). Chemical reference standards obtained from Sigma Aldrich were: ergosterol (Batch: MKCS7680), lanosterol, (Batch: L5768-1MG), and linoleic acid (Batch: 0000283282). 

#### 4.5.2. HPTLC Extraction Protocol

To prepare test portions for HPTLC fingerprinting, 75 mg (±1 mg) of sample powder was transferred into a 2 mL microcentrifuge tube. The fine powder was extracted in 1.5 mL pure methanol. The suspension was vortexed for 10 s, followed by sonication at room temperature for 15 min. The extracts were centrifuged at 7378× *g* for 10 min and the supernatant was transferred into HPTLC vials, for a final extract concentration of 50 mg sample mass per mL extraction solvent. 

Stock solutions of each standard were prepared by dissolving the compounds in methanol to appropriate concentrations. Standards were prepared at a concentration of 0.25 mg/mL in methanol.

#### 4.5.3. Chromatography System and HPTLC Fingerprint Analysis

The following HPTLC equipment and software from CAMAG (Muttenz, Switzerland) were used: Automatic TLC Sampler (ATS 4), Automatic Development Chamber (ADC 2), Scanner 4, Plate Heater III, TLC Visualizer 2, Immersion Device III, and VisionCats 4.0 software. Silica gel 60F_254_ HPTLC Premium Purity glass plates (20 × 10 cm) were manufactured by Merck (Darmstadt, Germany).

Aliquots were applied as narrow bands of 8.0 mm length at 8.0 mm from the lower edge of the plate. The bands of the outer lanes were applied 20 mm from the edge of the plate. The distance between tracks (center to center) was 11.0 mm. Application volumes were 10.0 µL. For identification testing, each chemical reference standard was applied at 2.0 µL. 

The mobile phase consisted of toluene, methanol, acetic acid 85:10:5 (*v*/*v*/*v*), with the development conditions as follows: pre-drying for 30 s, chamber temperature of 22 ± 5 °C, chamber saturation for 20 min, plate activation for 10 min at a relative humidity of 35% (±5%) using saturated MgCl_2_, preconditioning for 20 min, development to a distance of 70 mm, and drying for 5 min.

Anisaldehyde/sulfuric acid derivatization reagent was prepared fresh by mixing 20 mL of acetic acid with 170 mL of ice-cold methanol, followed by 10 mL of sulfuric acid, and then adding 1 mL of *p*-anisaldehyde. After development, plates were immersed in the derivatizing reagent (speed 5, time 0). Immediately afterward, the plates were heated at 100 °C for 3 min on a plate heater. The derivatized plates were then imaged under white light and 366 nm UV light.

### 4.6. UHPLC-ELSD/PDA Analysis of Triterpenoids, Sterols, and Fatty Acids

Acetonitrile, ethanol, formic acid used were of HPLC-certified grade, and water was purified using a Milli-Q system (Millipore, Bedford, MA, USA).

Authentic chaga samples were homogenized to a fine powder. Approximately 1000 mg of powdered chaga or commercial product contents were sonicated in 2.5 mL ethanol for 30 min, centrifuged at 10,000 rpm for 15 min, and supernatants pooled into a 5 mL volumetric flask. The extraction was repeated once, adjusted to final volume with ethanol, and filtered (0.22 µm) into LC vials.

All analyses were performed on a Waters H-class Acquity UPLC^TM^ system (Waters Corp., Milford, MA, USA), including a quaternary solvent manager, sample manager, column compartment, PDA and ELSD connected to Waters Empower 3 data station. An Acquity UPLC^TM^ C18 column also from Waters, was used for the separation. The column temperature was maintained at 50 °C. The mobile phase comprised: 0.1% formic acid in water (A), and 0.1% formic acid in acetonitrile (B). The gradient program, with a constant flow rate of 0.25 mL/min was used. Seven μL of the sample was injected, and peak identities were assigned based on the comparison of the retention times with standard compounds.

### 4.7. LC-DAD Triterpenoid Quantification

#### 4.7.1. Preparation of Test Solutions and Standards for LC-DAD Quantification

Chaga canker samples were sieved through a 40-mesh screen before analysis, while commercial products came as pre-milled fine powders. A 30 mg (±1 mg) portion sample powder was accurately weighed and transferred into 2 mL microcentrifuge tubes. The samples were extracted with 1.5 mL of pure methanol, followed by vortexing for 10 s and sonication at room temperature for 15 min. The extract was then centrifuged at 7378× *g* for 10 min, and the resulting supernatant was filtered through a 0.22 µm filter into LC vials, yielding a final extract concentration of 20 mg/mL. Reference standards were prepared in methanol, with inotodiol and trametenolic acid prepared at a concentration of 0.1 mg/mL, while 3ß-hydroxylanosta-8,24-dien-21-al and lanosterol were prepared at 0.05 mg/mL.

#### 4.7.2. HPLC-DAD Instrumentation and Chromatographic Conditions

Analyses were performed on an Agilent 1260 Infinity II Prime LC, controlled by OpenLab CDS 2.8. Separation was achieved on an Agilent Poroshell 120 EC-C18 column at 30 °C, with a sample injection volume of 10 µL. For calibration curve preparation, reference standards were injected at volumes between 1 and 7 µL, corresponding to different concentration levels. The mobile phase consisted of water (A) and acetonitrile (B). The gradient elution was carried out with a flow rate of 1.2 mL/min for the first 3.0 min, then increased to 1.6 mL/min from 3.0 to 6.0 min, while maintaining 100% B. A post-run wash step was performed, reducing % B from 100 to 70 between 6.0 and 7.2 min, followed by a hold at 70% B from 7.2 to 9.0 min at 0.8 mL/min. The % B was then increased back from 100% from 9.0 to 10.8 min, maintaining a flow rate of 0.8 mL/min. Finally, an equilibration step was applied from 10.8 to 14.5 min at 1.2 mL/min.

#### 4.7.3. Spectral Analysis and Compound Identification

Spectral analysis was performed to confirm the identity of the compounds in the chaga samples. UV-Vis absorbance spectra were recorded using an Agilent 1290 DAD FS, and the spectra of inotodiol, trametenolic acid, 3ß-hydroxylanosta-8,24-dien-21-al, and lanosterol reference standards were compared to corresponding peaks in the sample extracts. In all cases, the sample spectra matched the standards exactly, with identical absorption maxima.

#### 4.7.4. Calibration Curves, Quantification, and Method Validation

Quantification was performed by an Agilent 1290 DAD FS, with an absorbance detection wavelength set to 210 nm. Peak areas were integrated and compared to the four-point calibration curves to determine the concentrations of triterpenoids in the test solutions.

The method was validated according to ICH Q2(R2) guidelines, assessing accuracy, precision, specificity, linearity, and system suitability. The percentage recovery was assessed by spiking inotodiol (5.0, 10.0, and 15.0 µg, corresponding to 50%, 100%, and 150%) into the chaga voucher test solution, resulting in recoveries of 93.8%, 87.2%, and 89.5%, respectively. Intra-day and inter-day precision tests showed RSD values below 2%, demonstrating excellent repeatability and method reproducibility. For each triterpenoid, the limits of detection (LOD) and limits of quantification (LOQ) were determined using the standard deviation of the response (σ) and the slope of the calibration curve (S).

System suitability tests confirmed that peak resolution, retention time stability, and detector precision met acceptance criteria. Additionally, relative response factors (RRFs) were determined for triterpenoid quantification using inotodiol as the reference standard, allowing for a single calibration curve rather than separate ones for each triterpenoid.

### 4.8. ^1^H NMR Spectral Fingerprinting

#### 4.8.1. Sample Preparation for ^1^H NMR

Triplicate extractions were performed to account for variability. Finely ground chaga (0.19 *w*/*w* ratio to methanol) was vortexed, centrifuged (1480× *g*, 10 min), and 600 µL supernatant mixed with 100 µL CD_3_OD-d_4_ in 5 mm NMR tubes.

#### 4.8.2. Data Acquisition and Processing

Spectra were acquired on a Bruker Avance III HD spectrometer (400 MHz, 9.7 T) using a NOESY presaturation sequence for solvent suppression. Parameters:

I. Spectral width: 8223 Hz (20 ppm)
II. Scans: 64
III. Recycle delay: 15 s
IV. 90° pulse: Optimized per sample

Spectra were processed using Bruker Topspin^®^ software ver 3.6.3 for phasing, chemical shift referencing, and baseline calibration. Tetramethylsilane (TMS, 0 ppm) served as the chemical shift reference. 

### 4.9. LC QToF-MS Metabolomic Profiling

#### 4.9.1. Sample Extraction for LC QToF-MS

For whole dried chaga canker samples, the material was finely ground into a uniform powder. For pre-milled samples (commercial products), no additional grinding was necessary. Approximately 100 mg of each sample was weighed into 15 mL conical centrifuge tubes in duplicate. Each sample was extracted with 10 mL of 80:20 methanol/water (*v*/*v*) using a gentle mixing procedure on a rocking platform shaker for 48 h.

Following extraction, the samples were centrifuged at 1480× *g* for 10 min to separate the supernatant. The supernatant was filtered through 0.22 µm nylon syringe filters and transferred into LC vials for analysis.

#### 4.9.2. Instrumentation and Analysis

Separations were performed using an Agilent 1290 Infinity UHPLC (Agilent Technologies; Santa Clara, CA, USA) system equipped with an Agilent Zorbax Eclipse Plus C18 column (2.1 mm × 50 mm, 1.8 µm) and a guard column (3 mm × 5 mm, 1.8 µm). The column temperature was maintained at 40 °C. The mobile phase consisted of water (A) and methanol (B), and the flow rate was set to 0.200 mL/min. The injection volume was 1 µL. A gradient elution program was applied as follows: the mobile phase composition started at 75% B for 2.0 min, then increased linearly to 90% B over the next 4.0 min (2.0–6.0 min). From 6.0–8.0 min, the proportion of B further increased to 97%, where it was held for 32.0 min (8.0–40.0 min). Finally, the system was re-equilibrated at 75% B for 3 min before the next injection.

The Agilent 6546 QToF mass spectrometer was operated in positive ionization mode, with a capillary voltage of 3500 V, nozzle voltage of 250 V, fragmentor voltage of 140 V, and skimmer voltage of 65 V. The gas temperature was maintained at 300 °C, with a gas flow rate of 10 L/min and a nebulizer pressure of 35 psig. The sheath gas temperature was also set to 300 °C, with a sheath gas flow rate of 11 L/min.

#### 4.9.3. Data Processing

Mass spectrometric data were processed using software for peak detection, alignment, and normalization. Compounds were identified based on accurate mass measurements and isotopic distribution patterns, referencing a comprehensive database of fungal metabolites.

Compounds were imported into Mass Profiler Professional and grouped in two different interpretations for subsequent analysis. Upon grouping, the samples were explored using significance testing and fold change workflow. First, entities (compounds) are filtered to meet the frequency filter of being present in 100% of samples in at least one condition (group). This ensures that only compounds that are truly characteristic of the group are retained. Next, entities are filtered based on their *p*-values calculated from statistical analysis. The statistical test used was a one-way ANOVA with a *p*-value cut-off of 0.05. Finally, entities are displayed based on a fold change cut-off of 2.0 in at least one condition. A fold change of 2.0 represents a two-times increase in the intensity (quantity) of a given entity. 

PCA was used to visualize metabolomic differences between sample groups. Data were normalized and log-transformed before analysis. 

## 5. Conclusions

This study demonstrates the effectiveness of robust analytical methods for authenticating chaga (*I. obliquus*) dietary supplements. By integrating HPTLC, LC-ELSD, LC-QToF-MS, NMR spectroscopy, UV-Vis spectrophotometry, and iodine-starch assays, we identified critical chemical markers that distinguish wildcrafted chaga canker from fermented grain products and pure mycelium. This multi-analytical approach is novel in its application to authenticate chaga supplements, providing a comprehensive framework for quality control.

Authentic chaga canker was characterized by high melanin content, a high β-glucan to α-glucan ratio, and unique triterpenoid and phenolic profiles. In contrast, fermented grain products contained significant starch and α-glucans, lacked key triterpenoids, and exhibited metabolic profiles closely resembling their grain substrates rather than chaga canker. These findings emphasize a need for rigorous analytical verification to prevent misrepresentation in the marketplace.

Standardized multi-analytical approaches are essential for ensuring product authenticity, consumer confidence, and industry transparency. This framework can be applied to verify other functional fungal ingredients, setting a benchmark for quality control in dietary supplements.

## Figures and Tables

**Table 1:** UV-Vis absorbance and dilution factors for melanin content in chaga canker (WChV1-6), chaga canker 1:1 extract (ChExt11), *I. obliquus* mycelium (IoMyc1), and mycelia fermented grain samples at 500 nm. Dilution factors were applied to ensure that absorbance values remained within a measurable range for comparative analysis. WChV1-6 and ChExt11 exhibited vastly higher absorbance values, consistent with the behavior of melanin-like pigments. In contrast, IoMyc1 and IoMFG showed low absorbance, indicating negligible melanin-like pigments.

Composition	Sample Code	Absorbance	Dilution Factor
Whole Chaga Canker	WChV1	0.4768	2
	WChV2	0.6565	1
	WChV3	0.4854	2
	WChV4	0.5298	1
	WChV5	0.7053	2
	WChV6	0.7685	1
Chaga 1:1 Extract	ChExt11	0.6580	2
*I. obliquus* Mycelium	IoMyc1	0.0313	1
Fermented Grain	IoMFG-oat	0.0417	1
	IoMFG-unk	0.0492	1
	IoMFG-sorg	0.0457	1
	IoMFG-rice	0.0179	1

**Table 2:** Summary of triterpenoid quantification results in six whole chaga cankers (WChV1–6) and chaga canker 1:1 extract (ChExt11) using LC-DAD at 210 nm absorbance. The mean percentages (%*w*/*w*, dry) of inotodiol, trametenolic acid, 3β-hydroxylanosta-8,24-dien-21-al, and lanosterol are reported alongside relative response factors (RRF), standard deviation (SD), limit of detection (LOD), limit of quantification (LOQ), and R^2^ values for each compound. The data indicate that inotodiol is the most abundant triterpenoid.

Statistic	Inotodiol	Trametenolic Acid	3β-hydroxylanosta- 8,24-dien-21-al	Lanosterol
RRF	NA	1.28	1.39	1.43
Mean%	0.19	0.09	0.09	0.06
SD	0.080	0.022	0.030	0.017
LOD (ng)	19.9	16.7	8.4	3.0
LOQ (ng)	60.4	50.6	25.3	9.0
R2	1.0000	0.9998	0.9999	0.9999

## Data Availability

Data is contained within the article.

## References

[1] Kukulyanskaya T.A., Kurchenko N.V., Kurchenko V.P., Babitskaya V.G. (2002). Physicochemical properties of melanins produced by the sterile form of *Inonotus obliquus* (“Chagi”) in natural and cultivated fungus. Appl. Biochem. Microbiol..

[2] Allen E.A., Morrison D.J., Wallis G.W. (1996). Common Tree Diseases of British Columbia.

[3] Kahlos K. (1987). Studies on Triterpenes in Inonotus obliquus.

[4] Reid D.A. (1976). Inonotus obliquus (Pers. ex Fr.) Pilát in Britain. Trans. Br. Mycol. Soc..

[5] Glamočlija J., Ćirić A., Nikolić M., Fernandes Â., Barros L., Calhelha R.C., Ferreira I.C., Soković M., van Griensven L.J. (2015). Chemical characterization and biological activity of Chaga (*Inonotus obliquus*), a medicinal “mushroom”. J. Ethnopharmacol..

[6] Snell W.H., Dick E.A. (1971). A Glossary of Mycology.

[7] Gottesfeld L.M.J. (1992). Use of cinder conk (*Inonotus oblilquus*) by the Gitskan of the northwestern British Columbia, Canada. J. Ethnobiol..

[8] Shashkina M.Y., Shashkin P.N., Sergeev A.V. (2006). Chemical and medicobiological properties of chaga (review). Pharm. Chem. J..

[9] Szychowski K.A., Skóra B., Pomianek T., Gmiński J. (2020). *Inonotus obliquus*—From folk medicine to clinical use. J. Tradit. Complement. Med..

[10] Koyama T., Gu Y., Taka A. (2008). Fungal medicine, *Fuscoporia obliqua*, as a traditional herbal medicine: Its bioactivities, in vivo testing and medicinal effects. Asian Biomed..

[11] Kim Y.R. (2005). Immunomodulatory activity of the water extract from medicinal mushroom *Inonotus obliquus*. Mycobiology.

[12] Shen D., Feng Y., Zhang X., Liu J., Gong L., Liao H., Li R. (2022). In Vitro Immunomodulatory Effects of *Inonotus obliquus* Extracts on Resting M0 Macrophages and LPS-Induced M1 Macrophages. Evid. Based Complement. Alternat. Med..

[13] Song K.-C., Choi B.-L., Shin J.-W., Son J.-Y., Yoo H.-S., Cho J.-H., Lee Y.-W., Son C.-G., Cho C.-K. (2007). Effects of *Inonotus obliquus* extracts on immunomodulating activity. Kor. J. Ori. Med..

[14] Park Y.M., Won J.H., Kim Y.H., Choi J.W., Park H.J., Lee K.T. (2005). In vivo and in vitro anti-inflammatory and anti-nociceptive effects of the methanol extract of *Inonotus obliquus*. J. Ethnopharmacol..

[15] Ma L., Chen H., Dong P., Lu X. (2013). Anti-inflammatory and anticancer activities of extracts and compounds from the mushroom *Inonotus obliquus*. Food Chem..

[16] Alhallaf W., Perkins L.B. (2022). The anti-Inflammatory properties of chaga extracts obtained by different extraction methods against LPS-Induced RAW 264.7. Molecules.

[17] Ern P.T.Y., Quan T.Y., Yee F.S., Yin A.C.Y. (2023). Therapeutic properties of *Inonotus obliquus* (Chaga mushroom): A review. Mycology.

[18] Drenkhan R., Kaldmäe H., Silm M., Adamson K., Bleive U., Aluvee A., Erik M., Raal A. (2022). Comparative analyses of bioactive compounds in *Inonotus obliquus* conks growing on *Alnus* and *Betula*. Biomolecules.

[19] Hu H., Zhang Z., Lei Z., Yang Y., Sugiura N. (2009). Comparative study of antioxidant activity and antiproliferative effect of hot water and ethanol extracts from the mushroom *Inonotus obliquus*. J. Biosci. Bioeng..

[20] Nakajima Y., Sato Y., Konishi T. (2007). Antioxidant small phenolic ingredients in *Inonotus obliquus* (persoon) Pilat (Chaga). Chem. Pharm. Bull..

[21] Zheng W., Miao K., Liu Y., Zhao Y., Zhang M., Pan S., Dai Y. (2010). Chemical diversity of biologically active metabolites in the sclerotia of Inonotus obliquus and submerged culture strategies for up-regulating their production. Appl. Microbiol. Biotechnol..

[22] Maenaka T., Oshima M., Itokawa Y., Masubuchi T., Takagi Y., Choi J.S., Ishida T., Gu Y. (2008). Effects of Fuscoporia obliqua on postprandial glucose excursion and endothelial dysfunction in type 2 diabetic patients. J. Tradit. Chin. Med..

[23] Lu X., Chen H., Dong P., Fu L., Zhang X. (2010). Phytochemical characteristics and hypoglycaemic activity of fraction from mushroom *Inonotus obliquus*. J. Sci. Food Agric..

[24] Lee J.H., Hyun C.K. (2014). Insulin-sensitizing and beneficial lipid-metabolic effects of the water-soluble melanin complex extracted from *Inonotus obliquus*. Phytother. Res..

[25] Wang C., Chen Z., Pan Y., Gao X., Chen H. (2017). Anti-diabetic effects of *Inonotus obliquus* polysaccharides-chromium (III) complex in type 2 diabetic mice and its sub-acute toxicity evaluation in normal mice. Food Chem. Toxicol..

[26] Chung M.J., Chung C.K., Jeong Y., Ham S.S. (2010). Anticancer activity of subfractions containing pure compounds of Chaga mushroom (*Inonotus obliquus*) extract in human cancer cells and in Balbc/c mice bearing Sarcoma-180 cells. Nutr. Res. Pract..

[27] Géry A., Dubreule C., André V., Rioult J.P., Bouchart V., Heutte N., Eldin de Pécoulas P., Krivomaz T., Garon D. (2018). Chaga (*Inonotus obliquus*), a Future Potential Medicinal Fungus in Oncology? A chemical study and a comparison of the cytotoxicity against human lung adenocarcinoma cells (A549) and human bronchial epithelial cells (BEAS-2B). Integr. Cancer Ther..

[28] Kang J.H., Jang J.E., Mishra S.K., Lee H.J., Nho C.W., Shin D., Jin M., Kim M.K., Choi C., Oh S.H. (2015). Ergosterol peroxide from Chaga mushroom (*Inonotus obliquus*) exhibits anti-cancer activity by down-regulation of the β-catenin pathway in colorectal cancer. J. Ethnopharmacol..

[29] Kahlos K., Kangas L., Hiltunen R. (1986). Antitumour tests of inotodiol from the fungus *Inonotus obliquus*. Acta. Pharm. Finica.

[30] Nikitina S.A., Khabibrakhmanova V.R., Sysoeva M.A. (2016). Khimicheskiĭ sostav i biologicheskaia aktivnost’ triterpenovykh i steroidnykh soedineniĭ chagi [Composition and biological activity of triterpenes and steroids from *Inonotus obliquus* (chaga)]. Biomed Khim..

[31] Nomura M., Takahashi T., Uesugi A., Tanaka R., Kobayashi S. (2008). Inotodiol, a lanostane triterpenoid, from *Inonotus obliquus* inhibits cell proliferation through caspase-3-dependent apoptosis. Anticancer Res..

[32] Plehn S., Wagle S., Rupasinghe H.P.V. (2023). Chaga mushroom triterpenoids as adjuncts to minimally invasive cancer therapies: A review. Curr. Res. Toxicol..

[33] Shikov A.N., Pozharitskaya O.N., Makarov V.G., Wagner H., Verpoorte R., Heinrich M. (2014). Medicinal plants of the Russian Pharmacopoeia; their history and applications. J. Ethnopharmacol..

[34] Sun Y., Yin T., Chen X.H., Zhang G., Curtis R.B., Lu Z.H., Jiang J.H. (2011). In vitro antitumor activity and structure characterization of ethanol extracts from wild and cultivated Chaga medicinal mushroom, *Inonotus obliquus* (Pers.:Fr.) Pilát (Aphyllophoromycetideae). Int. J. Med. Mushrooms.

[35] Zhao F., Mai Q., Ma J., Xu M., Wang X., Cui T., Qiu F., Han G. (2015). Triterpenoids from *Inonotus obliquus* and their antitumor activities. Fitoterapia.

[36] Zhao Y., Zheng W. (2021). Deciphering the antitumoral potential of the bioactive metabolites from medicinal mushroom *Inonotus obliquus*. J. Ethnopharmacol..

[37] Kim J., Yang S.C., Hwang A.Y., Cho H., Hwang K.T. (2020). Composition of triterpenoids in *Inonotus obliquus* and their anti-proliferative activity on cancer cell lines. Molecules.

[38] Duru K.C., Kovaleva E.G., Danilova I.G., van der Bijl P. (2019). The pharmacological potential and possible molecular mechanisms of action of *Inonotus obliquus* from preclinical studies. Phytother. Res..

[39] Lu Y., Jia Y., Xue Z., Li N., Liu J., Chen H. (2021). Recent Developments in *Inonotus obliquus* (Chaga mushroom) polysaccharides: Isolation, structural characteristics, biological activities and application. Polymers.

[40] Wold C.W., Kjeldsen C., Corthay A., Rise F., Christensen B.E., Duus J.Ø., Inngjerdingen K.T. (2018). Structural characterization of bioactive heteropolysaccharides from the medicinal fungus *Inonotus obliquus* (Chaga). Carbohydr. Polym..

[41] Babitskaya V.G., Scherba V.V., Ikonnikova N.V., Bisko N.A., Mitropolskaya N.Y. (2002). Melanin complex from medicinal mushroom *Inonotus obliquus* 9Pers.: Fr.) Pilát (Chaga) (Aphyllophoromycetideae). Int. J. Med. Mushrooms.

[42] Peng H., Shahidi F. (2020). Bioactive compounds and bioactive properties of Chaga (*Inonotus obliquus*) mushroom: A review. JFB.

[43] Wu D.T., Deng Y., Chen L.X., Zhao J., Bzhelyansky A., Li S.P. (2017). Evaluation on quality consistency of *Ganoderma lucidum* dietary supplements collected in the United States. Sci. Rep..

[44] Beltrame G., Trygg J., Hemming J., Han Z., Yang B. (2021). Comparison of Polysaccharides Extracted from Cultivated Mycelium of *Inonotus obliquus* with Polysaccharide Fractions Obtained from Sterile Conk (Chaga) and Birch Heart Rot. J. Fungi.

[45] Lante A., Canazza E., Tessari P. (2023). Beta-Glucans of Cereals: Functional and Technological Properties. Nutrients.

[46] Henrion M., Francey C., Lê K.-A., Lamothe L. (2019). Cereal B-Glucans: The Impact of Processing and How It Affects Physiological Responses. Nutrients.

[47] Phuwadolpaisarn P. (2021). Comparison of β-Glucan Content in Milled Rice, Rice Husk and Rice Bran from Rice Cultivars Grown in Different Locations of Thailand and the Relationship between β-Glucan and Amylose Contents. Molecules.

[48] Wold C.W., Gerwick W.H., Wangensteen H., Inngjerdingen K.T. (2020). Bioactive triterpenoids and water-soluble melanin from *Inonotus obliquus* (Chaga) with immunomodulatory activity. JFF.

[49] Łysakowska P., Sobota A., Wirkijowska A. (2023). Medicinal Mushrooms: Their Bioactive Components, Nutritional Value and Application in Functional Food Production—A Review. Molecules.

[50] Sari M., Prange A., Lelley J.I., Hambitzer R. (2017). Screening of beta-glucan contents in commercially cultivated and wild growing mushrooms. Food Chem..

[51] McCleary B.V., Draga A. (2016). Measurement of β-Glucan in Mushrooms and Mycelial Products. J. AOAC Int..

[52] Rhee S.J., Cho S.Y., Kim K.M., Cha D.-S., Park H.-J. (2008). A comparative study of analytical methods for alkali-soluble ß-glucan in medicinal mushroom Chaga (*Inonotus obliquus*). LWT.

[53] Morris D.L. (1946). Colorimetric determination of glycogen; disadvantage of the iodine method. J. Biol. Chem..

[54] Quain D.E. (1981). The determination of glycogen in yeast. J. Inst. Brew..

[55] Brust H., Orzechowski S., Fettke J. (2020). Starch and Glycogen Analyses: Methods and Techniques. Biomolecules.

[56] Li X., Wu W., Zhang F., Hu X., Yuan Y., Wu X., Fu J. (2022). Differences between water-soluble and water-insoluble melanin derived from *Inonotus hispidus* mushroom. Food Chem. X.

[57] Ham S.S., Kim S.H., Moon S.Y., Chung M.J., Cui C.B., Han E.K., Chung C.K., Choe M. (2009). Antimutagenic effects of subfractions of Chaga mushroom (*Inonotus obliquus*) extract. Mutat Res..

[58] Upska K., Klavins L., Radenkovs V., Nikolajeva V., Faven L., Isosaari E., Lauberts M., Viksna A., Klavins M. (2023). Extraction possibilities of lipid fraction and authentication assessment of chaga (*Inonotus obliquus*). Biomass Conv. Bioref..

[59] Duan Y., Han H., Qi J., Gao J.-M., Xu Z., Wang P., Zang J., Liu C. (2022). Genome sequencing of *Inonotus obliquus* reveals inshights into candidate genes involved in secondary metabolite biosynthesis. BMC Genom..

[60] Papoutsis K., Grasso S., Menon A., Brunton N., Lyng J., Jacquier J.-C., Bhuyan D.J. (2020). Recovery of ergosterol and vitamin D2 from mushroom waste—Potential valorization by food and pharmaceutical industries. Trends Food Sci. Technol..

[61] Trinh T.A., Seo Y.H., Choi S., Lee J., Kang K.S. (2021). Protective Effect of Osmundacetone against Neurological Cell Death Caused by Oxidative Glutamate Toxicity. Biomolecules.

[62] Fordjour E., Manful C.F., Javed R., Galagedara L.W., Cuss C.W., Cheema M., Thomas R. (2023). Chaga mushroom: A super-fungus with countless facets and untapped potential. Front. Pharmacol..

[63] Hwang A.Y., Yang S.C., Kim J., Lim T., Cho H., Hwang K.T. (2019). Effects of non-traditional extraction methods on extracting bioactive compounds from chaga mushroom (*Inonotus obliquus*) compared to hot water extraction. LWT.

[64] Abu-Reidah I.M., Critch A.L., Manful C.F., Rajakaruna A., Vidal N.P., Pham T.H., Cheema M., Thomas R. (2021). Effects of pH and Temperature on Water under Pressurized Conditions in the Extraction of Nutraceuticals from Chaga (*Inonotus obliquus*) Mushroom. Antioxidants.

[65] Hao J., Wang X., Shi Y., Li L., Chu J., Li J., Lin W., Yu T., Hou D. (2023). Integrated omic profiling of the medicinal mushroom *Inonotus obliquus* under submerged conditions. BMC Genom..

[66] Zheng W., Zhang M., Zhao Y., Miao K., Pan S., Cao F., Dai Y. (2011). Analysis of antioxidant metabolites by solvent extraction from sclerotia of *Inonotus obliquus* (Chaga). Phytochem. Anal..

[67] Human Metabolome Dadatabse. https://hmdb.ca/.

[68] Uffelman C.N., Doenges K.A., Armstrong M.L., Quinn K., Reisdorph R.M., Tang M., Krebs N.F., Reisdorph N.A., Campbell W.W. (2023). Metabolomics Profiling of White Button, Crimini, Portabella, Lion’s Mane, Maitake, Oyster, and Shiitake Mushrooms Using Untargeted Metabolomics and Targeted Amino Acid Analysis. Foods.

[69] Megazyme® by Neogen. https://www.megazyme.com/beta-glucan-assay-kit-yeast-mushroom.

[70] Singla S., Htut K.Z., Zhu R., Davis A., Ma J., Ni Q.Z., Burkart M.D., Maurer C., Miyoshi T., Dhinojwala A. (2021). Isolation and Characterization of Allomelanin from Pathogenic Black Knot Fungus-a Sustainable Source of Melanin. ACS Omega.

